# Tracheotomy-coblation for acquired subglottic tracheal stenosis: a case report

**DOI:** 10.1186/s13019-019-0947-2

**Published:** 2019-07-04

**Authors:** Jingtao Huang, Zhongwei Zhang, Tao Zhang

**Affiliations:** Department of Thoracic Surgery, Tianjin Nankai Hospital, No. 6 Changjiang Road, Nankai District, Tianjin, 300100 China

**Keywords:** Acquired subglottic tracheal stenosis, Tracheotomy-coblation, Tracheal stenosis

## Abstract

**Background:**

Tracheal stenosis caused by tracheotomy and intubation is considered intractable. Although the segmental tracheal resection and endoscopic intervention are available, they usually result in great operation injury or are difficult to perform.

**Case presentation:**

A patient with acquired tracheal stenosis was treated with tracheotomy-coblation. The patient was followed up by bronchoscopy every 2 months. After 6-month follow-up, the symptoms of dyspnea and hoarseness disappeared and no tracheal stenosis was observed.

**Conclusions:**

The present technique, tracheotomy-coblation, is advantageous with less injury and easy to perform.

## Introduction

Acquired tracheal stenosis is considered as challenging due to difficult field visualization and instrument limitation. Coablation has advantages including rapid and precise ablation, little thermal damage, et al. However, more data is still needed to demonstrate the potential of coblation in managing airway stenosis [1]. Here, we report a novel technique using tracheotomy-coblation for treating subglottic tracheal stenosis that was resulted from post-intubation.

## Case presentation

**Fig. 1:**
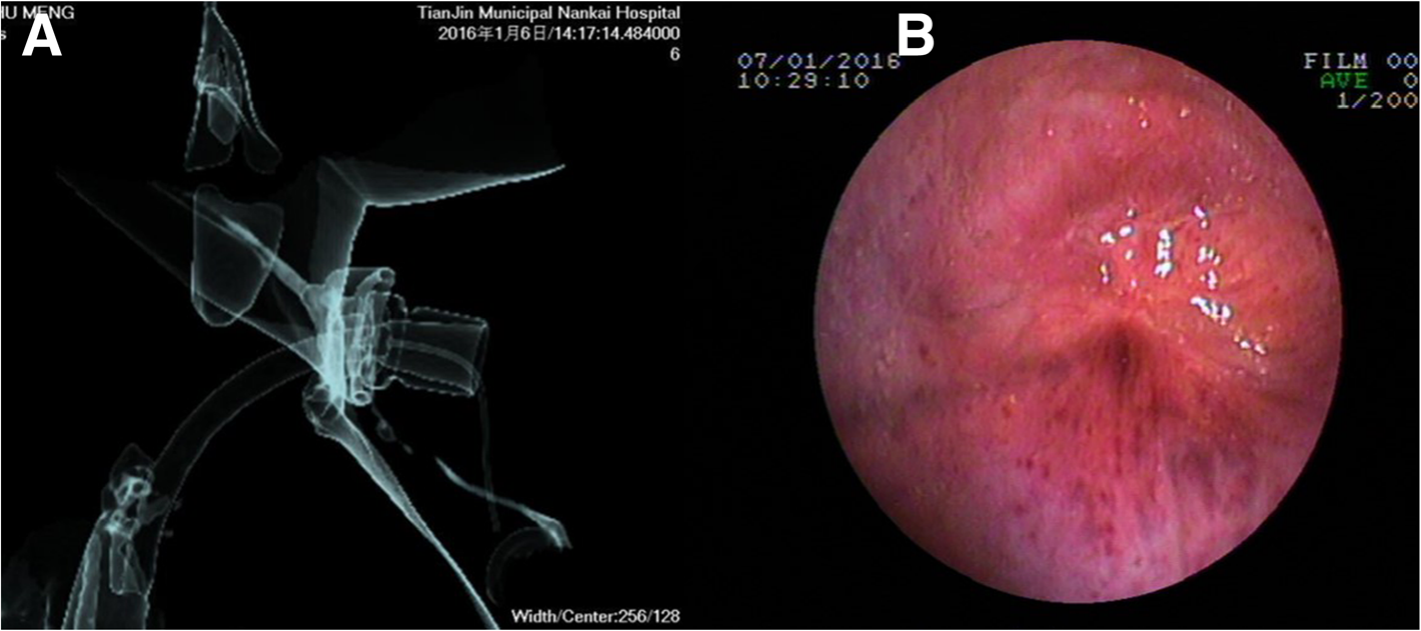
Preoperative examination. **a**: CT showed that the stenosis was 2 cm long. **b**: Bronchoscopy showed that the stenosis was at 2.5 cm below the glottis and the surface of the stenosis was completely epithelialized

This study was approved by the ethic committee of Tianjin Nankai Hospital. In February 2016, a patient (male, 26 years old) who fell from a great height and suffered from multiple fractures in the pelvic and legs underwent tracheal intubation and tracheotomy. Two months after, the tracheotomy catheter was removed and dyspnea occurred. Laryngoscopy showed granuloma hyperplasia combined with tracheal stenosis, and endotracheal intubation was performed by means of tracheostomy. Afterwards, the patient received several orthopedic surgeries, but the tracheal stenosis was left untreated. Five months prior to admission to our hospital, the patient was diagnosed as acquired tracheal stenosis and he refused multi-cryoablation therapy. According to computed tomography (CT) examination and bronchoscopy, it’s a 2 cm stenosis located at 2.5 cm below the glottis and the inner surface of the stenosis was smooth and completely epithelialized (Fig. 1). The mobility and appearance of the vocal cords were normal without obvious inflammation.

**Fig. 2:**
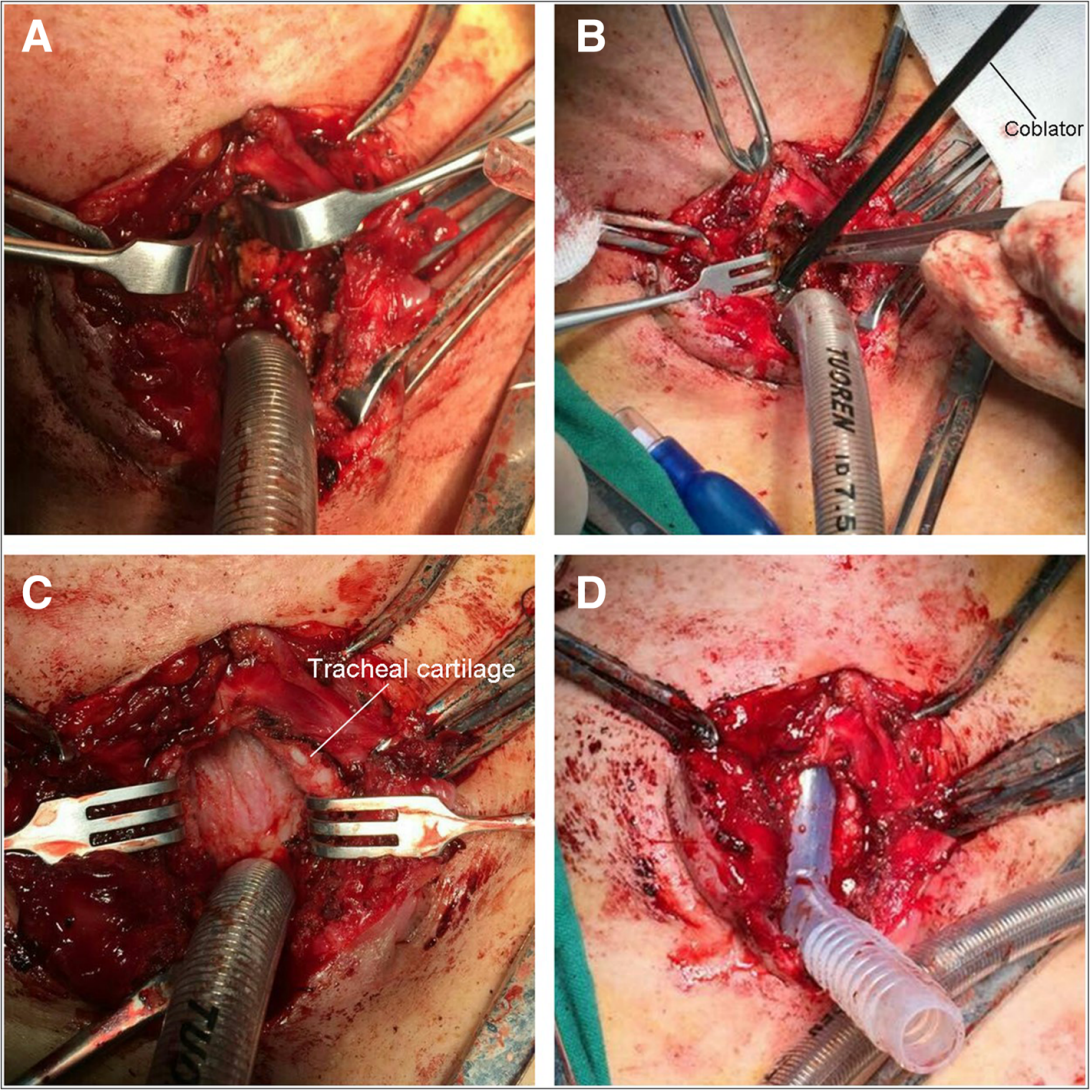
Operation procedures. **a**. The trachea was cut open to expose the anterior tracheal wall. **b**. The coblator was used to remove the tracheal scar. **c**. After coblation, the tracheal cavity was normal without obstruction. **d**. Placement of the Montgomery 16#T-tube

The patient was treated with tracheotomy-coblation; in trendelenburg’s position, the tracheal catheter was simply substituted by tracheal intubation under general anesthesia. The trachea was cut open to disassociate the anterior tracheal wall (2.5 cm-length). A syringe was used to puncture upward the anterior tracheal wall to determine the range of the stenosis. The coablation was performed with the tracheal cartilage as the anatomical landmark. After the trachea was cut open, the coblator (PLA-700 plasma surgery system, MECHAN Co. Ltd., Chengdu, China) was used to ablate the scar. A Montgomery 16#T-tube (Novatech, La Ciotat, France) was used and trimmed to cover the whole stenosis segment properly. The external diameter of the T-tube major branch was 16 mm and that of the collateral branch was 11 mm. The endotracheal catheter at the tracheotomy site was removed and the T-tube was implanted (Fig. 2). The tracheal intubation via the mouth was implemented for ventilation and the incision was closed. Bronchoscopy showed that the upper end of T-tube was at 1 cm below the glottis.

The tracheal catheter was successfully removed 2 h postoperatively, and the collateral branch of T-tube kept blocked. The patient had no difficulty in breathing, speaking and excreting sputum. Seven days postoperatively, the patient was discharged from our hospital and was hospitalized in an orthopedic hospital. The patient was followed up by bronchoscopy every 2 months.

Mild hoarseness occurred 12 months postoperatively. The hoarseness got worse and mild dyspnea occurred 15 months postoperatively. Bronchoscopy showed that the T-tube moved upward slightly with its upper end near the vocal cord. Significant congestion and edema as well as mild granuloma hyperplasia were observed in the glottis. We enlarged the incision under local anesthesia and removed the T-tube. The patient’s dyspnea and hoarseness were relieved. After 6-month follow-up, the symptoms of dyspnea and hoarseness disappeared and no tracheal stenosis was observed.

## Discussion

Subglottic tracheal stenosis resulted from tracheotomy is a very challenging condition. Traditional treatment includes segmental tracheal resection and endoscopic intervention. Traditional surgical resection and anastomosis has disadvantages, for example, the length of the resection. Besides, to avoid anastomotic stricture, the tracheal resection includes not only the atresia segment but also the abnormal segment of the stenosis and the stoma part. Thus sternotomy or manubriotomy was required [2]. Therefore, the surgical resection and anastomosis wasn’t considered as the optimal treatment.

Tracheal stenosis can also be treated through tracheal intervention [3], like laser, balloon dilatation, cryoablation, and tracheal stent. However, due to the stenosis, it’s difficult to perform the above treatments. It’s hard to precisely control the direction of ablation to avoid penetrating the tracheal wall and reaching the mediastinal space. Stenosis recurs easily after ablation. Secondly, the aging scar inside the trachea is difficult to ablate and requires repeated ablations to obtain satisfactory result if using cryoablation. Thirdly, using tracheal stent has the drawback of easy stent migration. The coablation has advantages including rapid and precise ablation, little thermal damage, and the integrated function of suction and coagulation [4]. Kitsko et al. [5] described the technique of coblation to remove a suprastomal granuloma in a single patient. However, more data is still needed to demonstrate the potential of coblation in managing airway stenosis, with only few reports up to now. Fastenberg et al. [1] reported its use in six pediatric otolaryngologic cases with different airway pathologies and Chan et al. [6] reported its use in adults with the airway stenosis less than 1 cm in length. This paper described tracheotomy-coblation in managing a 2 cm-long airway stenosis, and the result was satisfactory.

## Conclusion

The tracheotomy-coblation seemed advantageous with less operation injury compared to traditional surgery, and appeared to be safer and more convenient under direct visualization. It requires only a single operation which could simplify the tedious process of repeated ablation and reduce the patient’s burden.

## Data Availability

Please contact author for data requests.

## Abbreviation

CT: Computed tomography

## References

[1] Fastenberg JH, Roy S, Smith LP (2016). Coblation-assisted management of pediatric airway stenosis. Int J Pediatr Otorhinolaryngol.

[2] Elsayed H, Mostafa AM, Soliman S, Shoukry T, El-Nori AA, El-Bawab HY (2016). First-line tracheal resection and primary anastomosis for postintubation tracheal stenosis. Ann R Coll Surg Engl.

[3] Nouraei SA, Sandhu GS (2013). Outcome of endoscopic resection tracheoplasty for treating lambdoid tracheal stomal stenosis. Laryngoscope..

[4] Brown CS, Ryan MA, Ramprasad VH, Karas AF, Raynor EM (2017). Coblation of suprastomal granulomas in tracheostomy-dependent children. Int J Pediatr Otorhinolaryngol.

[5] Kitsko D, Chi D (2009). Coblation removal of large suprastomal tracheal granulomas. Laryngoscope.

[6] Chan CL, Frauenfelder CA, Foreman A, Athanasiadis T, Ooi E, Carney AS (2015). Surgical management of airway stenosis by radiofrequency coblation. J Laryngol Otol.

